# Everyday ethical attentiveness in stem cell-based model research

**DOI:** 10.1038/s44319-026-00945-8

**Published:** 2026-09-28

**Authors:** Nils Schütz

**Affiliations:** Max Planck School “Matter to Life” and Faculty of Theology, Heidelberg University, Heidelberg, Germany

**Keywords:** Development, Economics, Law & Politics, Science Policy & Publishing

## Abstract

The ethics of human stem cell-based models, from organoids to embryo models, has concentrated on the moral status and biological properties of research objects while overlooking everyday laboratory practices that shape and express ethical attitudes toward these objects before any formal definition or regulatory assessment.

**Figure:**
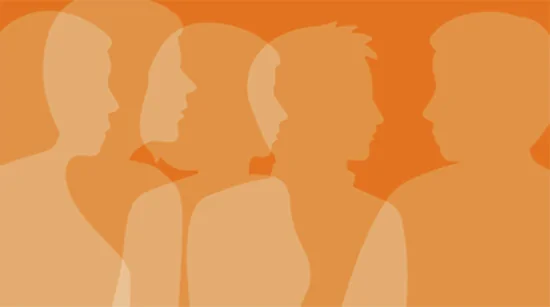

The ethics of stem cell-based research models is largely organized around regulation and governance. Organoids and embryo models are categorized for their supposed moral status, their biological properties, and how far research on them may proceed before it must stop. Regulation, however, can only be the second step. Before any research practices are assessed or any limits drawn, the ethical significance of doing research has to be registered. This first step happens in the context of everyday laboratory work, and it is also where practical ethics is primarily situated. An ethics that begins with a regulatory committee begins too late.

When Bredenoord, Clevers, and Knoblich first discussed the ethics of organoids, they wrote about donor consent, oversight of storage and use, and patient communication (Bredenoord et al, 2017). Subsequent reflections on the moral limits of organoid research focused on potential onset of consciousness in neural organoids (Koplin and Savulescu, 2019; Nuffield Council, 2026), moral status discussions (de Jongh et al, 2022), and chimeras (NASEM, 2021). Similarly, guidance on stem-cell-based embryo models proposed a set of biological attributes and developmental potentials as criteria by which oversight committees should assess proposals (Moris et al, 2026), along with stopping points based on developmental stage (De Los Angeles et al, 2026).

Across these documents runs a dominant tendency in how ethics is understood. According to this understanding, the task of ethics is to solve normative problems, which, conversely, means that only normative issues that are clearly identifiable and solvable can be the subject of ethics. This influences how research objects are evaluated ethically: the entity is examined for features of supposed moral relevance, its properties or developmental potentials are assessed, inadmissible practices are marked off, and the resulting limits are written into rules. Naturally, this kind of ethics has little to say about the ordinary conduct of research below the threshold of something that would be deemed problematic by any governance committee. After all, anything that cannot be framed as a morally dubious way of dealing with a research object is of little interest to a regulatory approach.

The attempt to infer the normative significance of research *practices* from supposedly evident morally relevant properties of research *objects* is what maintains this understanding, and it is this assumption that is in need of revision. To put it bluntly: Neither an organoid nor an embryo model carries an inherent moral demand within it. Moral demands are made by people who, depending on how they personally or professionally view these objects and relate to them, consider different ways of treating them to be fitting, careless, or inappropriate. A patient whose colorectal tumor is being studied in a patient-derived organoid relates differently to that culture than a lab technician testing the expected efficacy of targeted therapies. Similarly, a postdoctoral researcher whose career hinges on the next publication, a pharmaceutical company whose drug pipeline depends on organoid screening platforms, a tissue donor who learns that their material is being used commercially, and an activist convinced that organoids will end animal testing, may relate to the same (kinds of) research object. But these objects, as well as what they are used for, represent different things to these people or institutions, whose attitudes are not interchangeable.

An ethics that keeps searching research objects for features that would explain how they should be treated, but does not consider the social and practical contexts that led to their identification as something whose treatment should be questioned, is looking in the wrong place. This is why the laboratory is the primary site of ethically relevant practices: in how material is labeled, handled, stored, and discarded. This grants researchers no special authority over the ethical significance of their research; it identifies the site where that significance comes into play. The selection of issues that become the subject of governance and formal ethical deliberation is often already made, tacitly, in the routines that precede them. The laboratory is where many of these practices calling for ethical attentiveness are carried out, where the perspectives and interests of relevant parties are either taken into account or ignored, and where perception and reflection must be practiced and performed.

A 2023 paper by Landecker and Clark serves as a good example. They discuss the ethical relevance of naming a research object, and argue that describing embryo models as “synthetic human embryos” is inaccurate and disrespectful, and that the terminology shapes public understanding and regulation in problematic ways (Landecker and Clark, 2023). Ultimately, their criticism boils down to the argument that legitimate ways of speaking publicly about embryo models must reflect the specialized perspective of developmental biology. However, naming practices are never objective or neutral; they always represent a certain perspective. Someone who describes an embryo model as a “mere collection of cells” is speaking from a different standpoint than someone who refers to “test-tube babies.” Neither of these terms is, in and of itself, objectively problematic or unproblematic. The question is for which social context the naming convention is intended and whether it effectively communicates an instructive view about the entity in question.

A reorientation is already present in the field, albeit at its margins. Moos (2026) declines to assign moral status to laboratory entities and speaks instead of dignity-relevant practices. Sawai et al (2026) reach a neighboring position, using a relational ontology to recast status, personhood, and consent as matters of relation rather than intrinsic properties. Others who set out to move past the status question without shifting attention to practices have found it harder to leave behind. Barnhart and Dierickx (2023), for example, propose to move beyond the moral status of these entities, yet end up assuming a capability-based status position.

This leaves the question: What should be done? It is certainly not about replacing one checklist with another, going around the lab and scrutinizing every mundane task that is being performed. Instead, the goal is to train basic perception and reflection skills. In a lab, where people discuss the purpose of everyday routines now and again, inconsistencies stand out more clearly than when everyone is just working away on their own. Those who get into the habit of allowing themselves to acknowledge their own attitudes while conducting research are more likely to recognize the need for ethical clarification than those who keep their emotions at a distance. And recalling from time to time what inconspicuously named materials such as Matrigel or FBS are made of helps to avoid a potentially mindless use.

The value of such exchanges and exercises is not to produce moral problems where none exist, but to make one’s ethical perceptions answerable to others, to colleagues, to ethicists and potentially even to donors, publics and, eventually, regulators. Whether their cultivation requires trained ethicists, and whether the ethicist’s part is teaching, observing, participating, criticizing, or mediating, is an open question. Embedded ethics and practice-oriented research scholars have begun to explore it, but have yet to resolve it (Bentzen et al, 2026).

For more than a decade, the governance of stem cell-based research models has been dominated by an ethics of moral status and property discussions, and those have their uses. However, by focusing solely on research objects, these discussions and regulatory efforts lose touch with the habitual practices in the laboratory where stem-cell research actually takes place and where ethical relevance is first attributed. The more important ethical question is not what may be done to certain research models. It is whether the everyday conduct of this research can be noticed and become a subject of ethical inquiry.

## Supplementary information

Peer Review File
