# Supplementary material for: Everyday ethical attentiveness in stem cell-based model research
Source: EMBO Rep. 2026 Sep 28;27(20):5721–2. doi: 10.1038/s44319-026-00945-8 (PMC13694271; doi:10.1038/s44319-026-00945-8)
Supplement: Supplementary file 1 [file 44319_2026_945_MOESM1_ESM.pdf]

EMBO reports

Everyday ethical attentiveness in stem cell-based model research

Nils Schütz

Corresponding author(s): Nils Schütz (nils.schuetz@ts.uni-heidelberg.de)

Review Timeline:

Submission Date:	3rd Sep 26
Accepted:	15th Sep 26

Editor: Holger Breithaupt

Transaction Report: This article type is not peer reviewed.
